# Travelling Editorials

**Published:** 1843-09

**Authors:** 


THE WESTERN JOURNAL.

Vol. VIII.—No. III.

LOUISVILLE, SEPTEMBER 1, 1843.

### TRAVELLING EDITORIALS.

We wonder if any portion of the literature of the Augustan age was *brought forth* in August? If so, the *labour* must have been *tedious*, and the *offspring* half ASPHYXIATED. We presume that the books which have fallen *still born* from the press, were written in this month. To travel, and observe, and ask questions and make notes, and think, and indite editorials, in a moist and stagnant atmosphere, with the mercury at 90°, is positively too bad. We would earnestly advise all who look up to us for recipes to guide them in writing things fit to be read, to avoid the dog-days; in which advice we coincide with the great critic:

> "The dog-star rages, and beyond a doubt,
> All bedlam or Parnassus is let out."

We might, in addition to this fresh and erudite quotation, cite other authorities in support of our position, but prefer to establish it by the character of our editorials.

### *Goitre.*

Twelve months ago, we were wandering along the northern extremity of the Ohio and Erie canal, as we are now upon its southern. We there saw a number of cases of goitre, and heard of many more. Not a few of them were in their first stages, and the testimony of the physicians with whom we conversed, went to show that the malady is decidedly increasing in frequency. We find the same thing here. Indeed, within the last four or five years the cases in the valley of the Scioto have greatly multiplied. The patients are nearly all women in early life. Our brethren treat the disease with iodine, administered without and within; and, in general, speak encouragingly of its effects. On the remote causes of this malady, we shall not venture this muggy afternoon to offer any speculations; but in place of them, will respectfully solicit, from all who may read this article, whatever facts they can give us concerning the disease, and the geological conditions under which, any where in the west, they have observed it to occur.

*Return of Intemperance.*

The Washington temperance excitement has greatly abated in the southern part of Ohio. This is no more than might have been, and was, indeed, (by all discerning men) anticipated from the beginning; for all excitements, even political fermentations, religious revivals, and medical broils, are paroxysmal. Among the drunkards who reformed, we have met with some relapses, but the number is not great. They are sufficient, however, to afford an unenviable pleasure to those who did not change their habits, and to the cold-hearted and incredulous lookers-on, who, throughout the glorious reformation, found happiness in thinking and announcing that *they* had nothing to do with it. Should every intemperate man who has subscribed the pledge, within the last four years, return to the bottle, there would still remain to society benefits of sufficient value to render the excitement of those years, one of the most important social events of the present century. Wherever we have wandered for the last eighteen months, we have seen evidence of this, in modifications of manners and customs and habits which are likely to endure, and exert a permanent influence on the greatest vice of society. An example of this, is presented in a "Circular," designed for travellers, issued in 1841, by Mr. D. Talmadge, of Lancaster, Ohio, one of the most respectable stage proprietors of the West. From his nine "General Rules," we extract the following:

"Every driver addicted to drunkenness will be immediately discharged upon my becoming acquainted with the fact."

"Every driver is strictly prohibited the use of ardent-spirits, while driving upon his route, under penalty of being discharged at once."

Whoever saw such salutary regulations as these, in past times? We can all remember when a *majority* of stage drivers were addicted to drunkenness! Mr. Talmadge addresses a few words to passengers, some of which we are tempted to transcribe.

"A custom sometimes exists among passengers of inviting drivers to partake of a social glass, as an evidence of their approbation and respect, and often it is complied with from mere unwillingness to refuse. This thing though small in itself, may lead to the formation of a habit which will end in drunkenness, bringing ruin upon the unfortunate drivers, entailing misery and want upon his family. A trifling present, which would procure some small necessary of life for wife or children, would much better answer the purpose intended, while it would not form or strengthen a passion for strong drink."

Let no one smile or fret at our transcribing this paragraph. It embodies, in simplicity of language, a momentous principle—the influence of the higher upon the lower classes of society. As long as that influence is badly directed, intemperance, and the diseases of body and mind which it originates, will be kept up. Physicians are men of influence, and we call upon them. every where, to exert the moral power with which their profession has endowed them in the suppression of one of the most prolific causes of disease, which exists, or ever has existed, among men.

### *Medical Police of Jails.*

Every county jail in the United States, has its physician, but as yet these officials have done little, we believe, towards improving the cleanliness, ventilation, and police of these receptacles of the dissolute. Our attention has been turned upon this neglected, but not unimportant subject, by a sheet of printed "Rules for the Regulation and Government of the Jails in the Eighth Judicial District of Ohio," drawn up by its President Judge Hanna. By a late law of the State, the presiding judges are made the inspectors of the county jails, and authorized to direct their police. We shall copy from Judge Hanna's rules, such as relate more immediately to the health of the culprits, in the hope they may be adopted elsewhere:

"The Sheriff shall furnish each and every room, when occupied, with a broom, a bucket, pitcher or jug, containing pure wholesome water for drinking, and a wash bowl, bason, or other suitable vessel for washing the face and hands therein, with a sufficiency of soap and clean water for that purpose, a towel, and combs for the head; and shall require every prisoner to wash his or her face and hands, and comb his or her head, every morning before breakfast; also, to make his bed and sweep his room.

"He shall cause every prisoner having a beard, to be shaved at least once a week, and the hair of males to be cut as often as may be necessary for cleanliness and comfort.

"Every prisoner shall be required to change his, or her under-clothes, at least once a week.

"When any prisoner, except a debtor, has not a sufficiency of clothing to keep him, or her, comfortable and clean, it shall be the duty of the Sheriff to furnish the same, and cause the clothing of all prisoners (debtors excepted) to be washed and cleansed as often as may be necessary.

"Debtors shall in no case be confined with idiots, lunatics, insane or other prisoners. Prisoners charged with offences or crimes, shall not be confined with those convicted, males with females, colored with white prisoners, or youth with old offenders, where there are a sufficient number of rooms in the jail to admit of their separation.

"The Sheriff shall supply a bed-stead and bed with suitable clothing, for every two persons confined in jail, cause the sheets thereof to be changed and washed once a week, and the other clothes as often as necessary—the beds to be aired and sunned at least once a month, and where straw or chaff is used, cause the same to be emptied and filled with a fresh article at least once in six months; all the rooms to be sufficiently ventilated.

"No spirituous, malt, or fermented liquors, shall in any instance be allowed to any prisoner unless prescribed by the physician in actual attendance on the prisoner.

"The Sheriff shall at suitable times, at the request of any prisoner or prisoners, permit the clergy to visit the prison for instruction or religious exercise.

"He shall furnish each room, occupied by a prisoner capable of reading the same, with a copy of the Bible, and see that the same is not abused, injured, or destroyed."

The physician who is employed to attend on a prison, should not regard his duty as discharged by prescribing for the sick. Properly understood, it embraces an attention to every thing which can effect the health and reformation of the convict. But neither is to be promoted by personal and domestic filth, damp walls, intemperance, crowded cells, and foul air, against the whole of which the medical officer should enter a solemn protest. D.

*Portsmouth, Ohio,* August 1843.

